# Obinutuzumab for refractory minimal change disease in obese patients: a case series

**DOI:** 10.3389/fmed.2026.1842166

**Published:** 2026-05-14

**Authors:** Dandan Xue, Xiaofen Ma, Xiang Li, Yiming Zhang, Bing Xue

**Affiliations:** 1 Department of Nephrology, Affiliated Hospital of Jining Medical University, Jining, Shandong, China; 2 Department of Pathology, Affiliated Hospital of Jining Medical University, Jining, Shandong, China

**Keywords:** minimal change disease, obesity, obinutuzumab, refractory, rituximab

## Abstract

Minimal change disease (MCD) is a common cause of nephrotic syndrome in adults. Rituximab, a type I anti-CD20 antibody, is effective in many cases, but up to 40% of patients show an insufficient or transient response. Obesity, a frequent comorbidity, is associated with chronic low-grade inflammation and B-cell dysfunction, which may contribute to suboptimal treatment outcomes. Obinutuzumab, a type II anti-CD20 antibody, induces more profound B-cell depletion than rituximab. We report two obese patients (BMI 32.10 and 42.45 kg/m^2^) with biopsy-confirmed MCD who had an insufficient response to multiple immunosuppressive therapies, including rituximab. Patient 1 achieved complete remission with corticosteroids and tacrolimus; obinutuzumab (1 g) was added to enable rapid steroid withdrawal due to severe corticosteroid-induced acne. He remained in remission for >26 months after obinutuzumab, with CD19^+^ B-cell counts showing complete depletion (0 cells/μL), a rise to 103 cells/μL at 9 months (prompting a second dose). Patient 2 received obinutuzumab (1 g) for active disease after rituximab failure. He achieved complete remission but relapsed after immunosuppressant withdrawal. CD19^+^ B-cells had reconstituted to 75 cells/μL at relapse; a second obinutuzumab dose re-induced depletion (0 cells/μL) and remission, which was maintained at last follow-up. These observations suggest that obinutuzumab may induce and sustain remission in some obese patients with rituximab-insufficient MCD, and that obesity should be considered a potential modifier of treatment response in MCD.

## 1 Introduction

Minimal change disease (MCD) is the most frequent cause of idiopathic nephrotic syndrome in children and accounts for 10–15% of adult cases (1). It is a podocytopathy characterized by normal light microscopy and diffuse foot process effacement on electron microscopy (2, 3). Corticosteroids are first-line therapy; for steroid-resistant or frequently relapsing patients, calcineurin inhibitors, cyclophosphamide, and mycophenolate mofetil are used (4, 5). Rituximab, a type I chimeric anti-CD20 monoclonal antibody, has shown efficacy in refractory MCD (6). However, up to 40% of patients experience only transient responses or rapid relapse after rituximab, highlighting the need for more effective options (7).

Obesity (BMI ≥ 30 kg/m^2^) is a growing global health issue and a common comorbidity in adult MCD. It is increasingly recognized as an active modifier of immune function, inducing chronic low-grade inflammation and accelerated B-cell immunosenescence, with impaired protective antibody responses and expansion of pro-inflammatory B-cell subsets (8–10). These obesity-driven alterations could contribute to a more aggressive disease course and poorer response to rituximab, but the therapeutic implications of obesity in MCD have received little attention.

Obinutuzumab is a fully humanized, type II anti-CD20 antibody. Compared with rituximab, it is glycoengineered to enhance affinity for FcγRIIIa on effector cells, leading to superior antibody-dependent cellular cytotoxicity (ADCC) and direct cell death, while reducing dependence on complement-dependent cytotoxicity (CDC) (7, 11). These differences result in more profound and durable B-cell depletion, particularly of memory B cells that are often resistant to rituximab (12, 13). Given the distinct B-cell pathology induced by obesity, we hypothesized that obinutuzumab might be beneficial in obese MCD patients who had an insufficient response to rituximab. We here report two such patients.

## 2 Case description

### 2.1 Case 1

In April 2021, a 24-year-old obese male (body mass index 32.10 kg/m^2^) developed nephrotic syndrome. Renal biopsy confirmed minimal change disease, showing normal glomeruli on light microscopy and diffuse foot process effacement on electron microscopy. Initial laboratory findings included proteinuria 8.00 g/24 h, serum albumin 15.1 g/L, and serum creatinine 160.7 μmol/L (Table 1).

**Table 1:** Clinical course and laboratory parameters of Case 1.

Stage	Time range	Core therapy	24 h urinary protein (g)	Serum albumin (g/L)	Key changes in indicators
Initial treatment	April 2021	Methylprednisolone 60 mg daily	8.0 → 5.11	15.1 → 14.0	Urinary protein decreased, serum creatinine dropped sharply (elevated initially), while serum albumin remained severely reduced
Regimen adjustment + first remission	May 2021	Prednisone acetate 30 mg daily + Tacrolimus 2 mg q12h	5.11 → 2.0	14.0 → 37.3	Serum albumin returned to normal, urinary protein decreased significantly, and serum creatinine remained within the normal range persistently
Sudden abrupt rebound of indicators	Early June 2021	Methylprednisolone 40 mg daily + Tacrolimus 2 mg q12h	2.0 → 19.65	37.3 → 20.9	Urinary protein rebounded drastically to massive proteinuria, accompanied by a recurrent reduction in serum albumin
Rituximab intervention + rapid remission	Mid-June to July 2021	Rituximab 1 g × 2 doses + Tacrolimus dose reduction	19.65 → 0.62	20.9 → 40.5	Urinary protein plummeted, serum albumin restored to normal again, with a substantial improvement in all indicators
Mild rebound + hormonal fine-tuning	September to December 2021	Methylprednisolone/prednisone acetate + Tacrolimus 1.5 mg q12h	0.62 → 4.68 → 1.68	40.5 → 18.5 → 41.6	Mild rebound of urinary protein followed by a decline; serum albumin decreased transiently and then returned to normal
Deep remission + obinutuzumab intervention	May 2022 to April 2023	Tapering of prednisone acetate + Obinutuzumab 1 g × 2 doses + Tacrolimus maintenance	1.68 → 0.04 → 0.08 → 0.12	41.6 → 47.3 → 48.1 → 51.4	Urinary protein decreased to the normal range; serum albumin remained normal with a slightly elevated level, and serum creatinine was stable
Sustained stability after drug withdrawal	October 2023 to August 2024	No pharmacotherapy	0.12 → 0.06	51.4 → 47.3 → 45.3 → 48.8	Urinary protein remained at a low normal level; serum albumin and serum creatinine were stably maintained within the normal range throughout the period

High-dose corticosteroids were initiated, and tacrolimus was added (trough levels maintained at 7-10 ng/mL). The patient developed severe facial acne as an adverse reaction to corticosteroids, which was poorly responsive to topical and systemic treatments. Rituximab was then administered to enable steroid tapering, but the patient relapsed after dose reduction, prompting reintroduction of corticosteroids and tacrolimus.

By May 30, 2022, complete remission was achieved (proteinuria 0.04 g/24 h, albumin 47.3 g/L, creatinine 68.7 μmol/L). However, because of persistent corticosteroid-induced acne, obinutuzumab 1 g was given on July 5, 2022, together with tapering tacrolimus and prednisone, in order to maintain remission while rapidly discontinuing corticosteroids.

Peripheral blood CD19^+^ B-cell counts were monitored as follows:

- July 5, 2022 (day of obinutuzumab): 0 cells/μL.
- December 26, 2022: 0 cells/μL.
- April 6, 2023: 103 cells/μL → prompting a second obinutuzumab 1 g dose on the same day.
- October 18, 2023: 0 cells/μL.
- January 24, 2024: 1 cell/μL.

Despite B-cell reconstitution, the patient remained in complete clinical remission. Corticosteroids were successfully discontinued after obinutuzumab treatment. At the last follow-up (August 2024), proteinuria was 0.06 g/24 h, albumin 48.8 g/L, and creatinine 64.5 μmol/L, with all immunosuppressive therapy discontinued. The remission duration after the first obinutuzumab dose exceeded 26 months.

Safety: Neutrophil counts remained within the normal range, and no adverse reactions were observed.

### 2.2 Case 2

In March 2020, a 28-year-old severely obese male (BMI 42.45 kg/m^2^) was admitted with nephrotic syndrome. Renal biopsy confirmed minimal change disease (MCD). Initial laboratory findings: proteinuria 9.63 g/24 h, albumin 12.9 g/L, creatinine 75.8 μmol/L (Table 2).

**Table 2:** Clinical course and laboratory parameters of Case 2.

Stage	Time range	Key treatment events	24 h urinary protein (g)	Therapeutic response & indicator changes
Initial treatment	Mar – Aug 2020	Tacrolimus initiated and dose-adjusted; Prednisone 50 mg added due to worsening condition	9.63 → 8.84 → 7.29 → 18.64	Tacrolimus monotherapy failed to control disease progression, presenting with elevated Scr, increased massive urinary protein, and persistent severe hypoalbuminemia. Adding prednisone did not reverse progression.
First remission induction	Feb – Sep 2021	First Rituximab (2.5 g); methylprednisolone 80 mg pulse therapy for relapse; second Rituximab (1 g)	12.84 (relapse) → 0.75 → 0.30	First rituximab (2.5 g) achieved partial remission, but relapse occurred with increased urinary protein and aggravated hypoalbuminemia. Methylprednisolone 80 mg pulse therapy plus a second rituximab dose (1 g) induced near-complete remission, with urinary protein ≤0.30 g/24 h and Alb normalized.
Relapse & rescue treatment	Oct 2021 – Mar 2022	Relapse after steroid/tacrolimus taper; severe relapse occurred; pulse therapy; first Obinutuzumab (1 g); switch to Cyclosporine	0.30 → 7.18 (relapse) → 18.23 (severe relapse) → 0.21 → 0.60	Immunosuppressant tapering triggered relapse, progressing to severe massive proteinuria. Pulse therapy induced rapid remission; switching to cyclosporine plus first obinutuzumab (1 g) consolidated remission, with stable favorable indicators.
Immunosuppressant withdrawal & catastrophic relapse	Oct 2022 – Jan 2023	All immunosuppressants discontinued; catastrophic relapse; second Obinutuzumab (1 g)	0.60 → 27.56 (catastrophic relapse) → 17.91	Complete immunosuppressant withdrawal caused catastrophic relapse, with urinary protein surging to 27.56 g/24 h and severe hypoalbuminemia recurring. Notably, Scr remained normal (no acute renal injury). A second obinutuzumab dose (1 g) only partially reduced urinary protein by January 2023.
Long-term follow-up after relapse	May 2024 – Jul 2025	No specified immunosuppressive therapy	Not detected	During long-term follow-up, Alb remained stably normal, and Scr was normal with mild fluctuations. Urinary protein was undetected; however, given prior catastrophic relapse, spontaneous remission or residual antibody effects may account for disease control.

Treatment with tacrolimus (trough 9–10 ng/mL) and subsequent prednisone failed to control the disease. Rituximab (2.5 g, then 1 g) induced only a transient response, with rapid relapse after dose tapering.

On March 26, 2022, obinutuzumab 1 g was administered for the first time, together with cyclosporine and prednisone. Cyclosporine trough levels were not monitored (routine drug monitoring not performed at our center at that time). The patient achieved complete remission, and corticosteroids were gradually tapered. As of October 20, 2022, the CD19 + B-cell count remained at 0 cells/μL.

However, on December 22, 2022, the patient experienced a catastrophic relapse (proteinuria 27.56 g/24 h, albumin 14.3 g/L). The CD19 + B-cell count had risen to 75 cells/μL. A second dose of obinutuzumab 1 g was given immediately. On January 6, 2023, CD19 + B-cell count was 0 cells/μL; on July 26, 2023, it was 3 cells/μL. Proteinuria gradually became undetectable.

Safety: Neutrophil counts remained within the normal range, and no adverse reactions were observed.

## 3 Discussion

We report two obese patients with biopsy-confirmed MCD who had an insufficient response to rituximab. Patient 1 received obinutuzumab to facilitate steroid withdrawal after achieving remission; Patient 2 received it for active disease. Both achieved long-term remission, although Patient 2 relapsed once after B-cell reconstitution (CD19^+^ 75 cells/μL) and required re-treatment.

A significant element of MCD pathogenesis is immune dysregulation, characterized by T-cell anomalies and the formation of autoantibodies against podocyte antigens such as nephrin (14, 15). By binding directly to nephrin, these autoantibodies can cause its dysfunction or removal from the slit diaphragm, aggravating breakdown of the filtration barrier and contributing to nephrotic syndrome. According to Bertelli et al., MCD pathogenesis involves a complex immune model activating innate immunity, APCs, and B cells, leading to production of oxidative species (16). While the exact mechanisms remain incompletely understood, there is increasing focus on the autoimmune elements of MCD, implicating B cells as key players.

The differential response to rituximab and obinutuzumab observed in our obese patients may be explained by the profound impact of obesity on B cell biology, which creates a unique therapeutic challenge and a specific niche for type II anti-CD20 therapy.

Several recent case reports and small series have described the successful use of obinutuzumab in rituximab-refractory MCD and focal segmental glomerulosclerosis (FSGS) (17–21). Our findings are consistent with these reports and extend the literature by specifically focusing on obese patients.

Obesity induces accelerated B-cell immunosenescence, including leptin-mediated inflammation, expansion of pro-inflammatory double-negative B cells, and metabolic adaptation that may compromise responses to rituximab (8, 22–24). Obinutuzumab, with enhanced ADCC and direct cell death, might theoretically overcome some of these barriers (7, 11, 13). However, our data do not prove this mechanism; it remains speculative.

These observations have several clinical implications. First, obesity may serve as a key modifier of treatment response in MCD, and should be considered when selecting anti-CD20 therapy. For obese patients with highly relapsing disease, early transition to obinutuzumab may be warranted. Second, the success of obinutuzumab in this challenging population suggests that type II anti-CD20 antibodies may be particularly suited for conditions characterized by pro-inflammatory B cell expansions, such as obesity-associated autoimmunity.

Future research should prospectively compare the efficacy of obinutuzumab versus rituximab in obese versus non-obese MCD patients, with stratification by BMI. Mechanistic studies examining B cell subsets (e.g., DN B cells), adipokine profiles (e.g., leptin), and metabolic parameters before and after treatment could identify biomarkers predictive of response and provide further insights into the interplay between obesity and B cell pathology in MCD.

### 3.1 Limitations

- Retrospective, uncontrolled case series (*n* = 2).
- Concomitant immunosuppressants prevent attributing efficacy solely to obinutuzumab.
- No biomarker or B-cell subset analysis.
- Small sample size precludes generalization.

## 4 Conclusion

Obinutuzumab induced and sustained complete clinical remission in two obese patients with rituximab-refractory MCD, with profound B-cell depletion documented. The enhanced B-cell depleting capacity of obinutuzumab may be particularly advantageous in the setting of obesity-related B-cell dysregulation. Obesity should be considered a key modifier of treatment response in MCD, and obinutuzumab represents a promising therapeutic option for this challenging subgroup.

## 5 Patient perspective

Both patients provided written informed consent for publication. Patient 1, a 24-year-old man, expressed great relief after achieving stable remission. He particularly appreciated that obinutuzumab allowed rapid tapering and discontinuation of corticosteroids, which resolved the severe facial acne that had significantly affected his social life. He reported returning to full-time employment and normal daily activities. Patient 2, a 28-year-old man, initially felt frustrated by repeated relapses and the burden of frequent hospital visits. After the second obinutuzumab dose and sustained remission, he regained confidence in disease control and reported improved quality of life. Both patients were satisfied with the anonymity of their clinical data.

## Data Availability

The original contributions presented in the study are included in the article/supplementary material, further inquiries can be directed to the corresponding authors.
